# Acetylcysteine and budesonide for the treatment of refractory Mycoplasma pneumoniae pneumonia in children: a clinical observation

**DOI:** 10.1186/s13052-023-01491-y

**Published:** 2023-07-08

**Authors:** Yanli Wang, Kai Liu, Chuangui Chen, Chengyun Zhang

**Affiliations:** 1 Department of Pediatrics, South China Hospital, Medical School, Shenzhen University, Guangdong, China; 2 Pulmonary and Critical Care Medicine, Kunming Children`s Hospital, Kunming, China; 3 Department of Pediatrics, The First People’s Hospital of Yunnan Province, Kunming, China; 4 Department of Pediatric, The First People’s Hospital of Zhengzhou City, Zhengzhou, China

**Keywords:** Refractory *Mycoplasma pneumoniae* pneumonia, Acetylcysteine, Bronchoscopic alveolar lavage, Children

## Abstract

**Background:**

To examine the clinical impact of bronchoscope alveolar lavage (BAL) combination with budesonide, ambroxol + budesonide, or acetylcysteine + budesonide in the treatment of refractory Mycoplasma pneumoniae pneumonia (RMPP).

**Methods:**

Eighty-two RMPP patients admitted to Pediatrics at The First People’s Hospital of Zhengzhou were retrospectively evaluated between August 2016 and August 2019. All patients were administered BAL in addition to intravenous Azithromycin, expectoration, and nebulizer inhalation. The medications added to the BLA separated the patients into the Budesonide group, Ambroxol + budesonide group, and acetylcysteine + budesonide group. Analyzed were the variations in laboratory examination indices, improvement in lung imaging, overall effective rate, and adverse responses in the three groups.

**Results:**

The laboratory test indices of patients in all three groups improved significantly relative to pre-treatment levels, and the results were statistically significant. After therapy, there were no significant differences between the three groups in terms of white blood cell (WBC), C-reactive protein (CRP), or erythrocyte sedimentation rate (ESR). Serum lactate dehydrogenase (LDH) and serum ferritin (SF) varied significantly across the three groups (P < 0.05). In the acetylcysteine + budesonide group, the absorption rate of lung imaging lesions and clinical efficacy were superior to those of the other two groups. There were no significant differences between the three groups in the occurrence of adverse events (P > 0.05).

**Conclusions:**

BLA-coupled acetylcysteine + budesonide was superior to the other two groups in enhancing the effectiveness of RMPP in children, which might increase lung opacity absorption and minimize lung inflammation.

## Background

Mycoplasma pneumoniae (MP) is the most common agent responsible for community-acquired pneumonia (CAP) in children [1]. Cases of severe or refractory Mycoplasma pneumoniae pneumonia (MPP) have been documented worldwide, mainly in Asia[2]. Traditionally, macrolides have had a more significant therapeutic effect. In some pediatric cases, however, the infection may progress to a form resistant to conventional macrolide treatment [3, 4]. Bronchoscopy is a significant revolution in treating respiratory problems; it was first used in 1978[5] on children. Bronchoalveolar lavage (BAL) is an applicable diagnostic and therapeutic procedure for pediatric respiratory diseases caused by immunology, inflammation, and infection [6–8]. Bronchoscopy enables the segmental or subsegmental bronchus to be rinsed or injected with medicines [9]. BLA may efficiently remove airway secretions and dilute the bronchial lumen secretions. BAL is used to treat refractory mycoplasma pneumonia in children. Stimulating the local airway mucosa improves the patient’s sputum excretion and facilitates the discharge and suction of inflammatory secretions.

In contrast, the local injection of therapeutic drugs increases the local concentration of drugs in the bronchi, reduces lung inflammation, and enhances the therapeutic effect. In China, amiloride injection, saline, budesonide nebulizer, and acetylcysteine nebulizer are the most regularly used medications for the lavage solution during bronchoscopic alveolar lavage, with fewer reports coming from other countries. There is no standard clinical procedure for choosing remedies for RMPP lavage fluid. This study investigated the effect of several lavage fluids on treating RMPP during bronchoscopy for clinical reference.

## Materials and methods

### Study subjects

Research subjects From August 2016 to August 2019, children with RMPP were recruited from the Department of Pediatrics at The First People’s Hospital in Zhengzhou, China, According to the diagnostic criteria of RMPP [3]: (1) the fever persisted, and clinical symptoms deteriorated after seven days of anti-infective therapy with macrolides. (2) Deterioration of lung imaging signs; imaging manifestations: lobar pneumonia, bilateral lung inflammation, atelectasis, consolidation, pleural effusion, etc. (3) Diagnosis of MP infection: the IgG titer of MP Antibody(Ab) in two serum samples rose more than fourfold within four weeks. One serum MP Antibody IgM is positive, and the nasopharyngeal swab or bronchoalveolar lavage fluid MP PCR DNA is positive. The MP infection may be created if two of them are positive. With the following conditions excluded: (1)Immunodeficiency illness, cardiovascular disease, recurrent respiratory infection, bronchopulmonary dysplasia, and bronchiolitis obliterans. (2)Patients who are long-term users of immunosuppressive medications. (3)Patients with infections caused by Mycobacterium TB, adenovirus, syncytial virus, rhinovirus, fungi, bacteria, and other pathogens. Positive serum MP antibody IgM was detected in 82 children through serological testing. Among these cases, 69 patients tested positive for MP DNA through PCR on nasopharyngeal swabs, while 76 patients tested positive for MP PCR DNA on bronchoalveolar lavage fluid through bronchoalveolar lavage. This research was authorized by the Ethics Committee of the First People’s Hospital of Zhengzhou, and all patients’ guardians provided informed permission upon admission. All patients had a bronchoscopic evaluation of the alveoli and were split into three groups based on the administration of budesonide in the lavage fluid: budesonide (n = 24), ambroxol + budesonide (n = 28), and acetylcysteine + budesonide (n = 30).

### Methods

#### Retrospective analysis

Retrospective investigation 82 children diagnosed with RMPP were treated with bronchoscopic alveolar lavage. Electronic medical records were used to retrospectively examine clinical patient data, including gender, age, duration of stay, etc. On the morning of the second day after admission, all patients had a complete blood count, C-reactive protein (CRP), erythrocyte sedimentation rate (ESR), lactate dehydrogenase (LDH), and serum ferritin (SF). All the children received an intravenous injection of Azithromycin (10 mg/kg/day) for 7 to 10 days to treat the MP infection, along with cough medication and expectorant. For certain patients with MP complicated by bacterial infection, second or third-generation cephalosporins were used to combat bacterial infections. Injections of methylprednisolone (2 mg/kg/d, twice daily) were administered to patients with fast disease development. Before and after BLA with various medications, the changes in laboratory indicators, the improvement rate of pulmonary iconography, the improvement rate of clinical symptoms, and the incidence of side effects were studied.

#### BLA methods

BLA techniques Eighty-two individuals received Bronchoscopy examination and BLA; bronchoscopy requirements carried out preoperative preparations, and all patients fasted for 6 to 8 h before bronchoscopy. The Olympus BF-P26F and BF-X26F bronchoscopes were used to assess and treat the youngsters. Different diameters of bronchoscopy were chosen based on the age of the kid. Over one year old, Olympus BF-P26F bronchoscopes were used, whereas Olympus BF-XP26F bronchoscopes were utilized under one year. Before BLA, lidocaine 2 ml and ipratropium bromide 1 ml atomizing solution was administered. It was injecting 2% lidocaine into the nasal cavity. 2–4 L/min of oxygen was breathed, and 0.3 mg/kg of midazolam was steadily delivered intravenously. The patient was administered 2% lidocaine(1ml) by “anesthesia on entrance” to the glottis, trachea, left and right bronchus, with the total dose not exceeding 7 mg/kg. BLA was conducted based on lung imaging findings of inflammation or atelectasis. The BLA fluid (BLAF) was randomly selected. BLAF preparation procedures: Normal saline 20ml + Budesonide 2 ml, Normal saline 20 ml + Budesonide 2 ml + Ambroxol Hydrochloride Injection 15 mg, or Normal saline 20 ml + Budesonide 2 ml + Acetylcysteine Solution 6 ml. The maximal capacity of each lavage was no more than 5ml/kg. BALF gathers 100 to 200 mmHg of negative pressure. The BALF recovery rate was between 60 and 80%. Before lavage, the bronchoscope wall should be cleansed and brushed using a bronchoscope scrub if it is difficult to remove secretions that have adhered to the bronchial wall. Blood oxygen saturation, heart rate, respiration, complexion, and circulation were continually monitored during the surgery. Depending on the patient’s hospital status, BLA was done one to three times. BLA fluid was extracted.

### Outcome measures

#### Clinical general therapeutic effect

The therapy impact was assessed based on lung symptoms, indicators, CT scan findings, etc. The following criteria [1, 10]: (1) Remarkable effect: fever, cough, expectoration, and other clinical symptoms vanished, respiratory sounds and body temperature returned to normal, lung rales vanished, and lung imaging revealed that the inflammation had been entirely absorbed. (2) Effective: cough, expectoration, and shortness of breath symptoms decreased, body temperature returned to normal, lung rales diminished, and breath sounds improved. Imaging of the lungs revealed a partial absorption of inflammation. (3) Ineffective: cough, expectoration, and shortness of breath were not alleviated or worsened, body temperature was average or increasing, and lung imaging revealed inflammation was not absorbed. Below is the calculating formula:

### Statistical analysis

Statistical examination SPSS 19.0 was used for data analysis. Data are represented as the mean ± standard deviation. A paired t-test was conducted before and after therapy. A one-way ANOVA was used to compare the groups. The Chi-square test was used for data counting.

## Results

### General information

As indicated in Table 1, there was no significant difference between the three groups regarding age and gender (F = 0.565, P > 0.05). The duration of hospital stays varied significantly across the three groups (F = 3.61, P < 0.05). The length of hospitalization was shorter in the acetylcysteine + budesonide group compared to the other two groups, but there was no significant difference between the other two groups, P > 0.05. Imaging findings did not vary significantly across the three groups. The difference between the number of lavages in the acetylcysteine + budesonide group and the other two groups was statistically significant.

**Table 1:** General data of 82 children with RMPP

	Budesonide	Ambroxol + Budesonide B	Acetylcysteine + Budesonide	Statistical results	
	n = 24	n = 28	n = 30		
**Average age (years)**	5.70 ± 2.25	5.80 ± 2.58	5.18 ± 2.33	* F* = 0.565	*P* = 0.571
**Gender(**male/ female)	13/11	14/14	16/14	*X*^*2*^ = 0.105	*P* = 0.949
**hospital stay (days)**	13.33 ± 2.16	12.93 ± 2.18	12.65 ± 2.22	* F* = 3.610	*P* = 0.032
**Pulmonary imaging**					
Unilateral lesion of a lobe or segment	16	20	21	*X*^*2*^ = 0.073	*P* = 0.964
Bilateral lesions of a certain lobe or segment	8	8	9		
pleural effusion	8	9	11	*X*^*2*^ = 0.144	*P* = 0.931
atelectasis	9	10	12	*X*^*2*^ = 0.114	*P* = 0.944
pulmonary consolidation	1	3	4	*X*^*2*^ = 1.317	*P* = 0.518
**MP detection**					
MP-IgM Ab(+)	12	13	13	*X*^*2*^ = 0.238	*P* = 0.888
MP-IgG Ab(≥ 1:160)	17	21	23	*X*^*2*^ = 0.246	*P* = 0.884
MP-PCR DNA(+)	19	22	24	*X*^*2*^ = 0.018	*P* = 0.991
Both two items were positive simultaneously	21	25	27	*X*^*2*^ = 0.088	*P* = 0.957
Both three items were positive simultaneously	3	3	3	*X*^*2*^ = 0.088	*P* = 0.957
**Therapeutic drugs**					
Azithromycin + methylprednisolone	12	13	13	*X*^*2*^ = 2.705	*P* = 0.951
Azithromycin + methylprednisolone + cefuroxime sodium injection	6	6	5		
Azithromycin + methylprednisolone + ceftriaxone sodium injection	3	4	6		
Azithromycin + methylprednisolone + piperacillin-tazobactam	2	4	4		
Azithromycin + methylprednisolone + cefoperazone sulbactam	1	1	2		
Number of **BLA**					
One time	18	22	27	*X*^*2*^ = 2.870	*P* = 0.058
Two times	4	5	2		
Three times	2	1	1		
**Selection of bronchoscope diameter**					
diameter 4.0 mm	23	26	29	*X*^*2*^ = 0.490	*P* = 0.783
diameter 2.8 mm	1	2	1		

### Comparison of laboratory examination indicators

After BLA was treated with various medicines, the changes in laboratory examination indices in the three groups before and after therapy were statistically significant (P < 0.01). Laboratory examination indices did not vary significantly across the three groups. Before and after hospitalization, there were substantial differences between the three groups in LDH and SF. (Table 2).

**Table 2:** Laboratory examination indexes of 3 groups of children with RMPP

Laboratory indexes	Group	Cases	Before treatment	After treatment	*t*	*P*
CRP (mg/dl)	Budesonide	24	56.73 ± 43.98	13.98 ± 9.06	5.49	< 0.001
	Ambroxol + budesonide	28	49.99 ± 41.50	7.92 ± 4.69	5.85	< 0.001
	Acetylcysteine + budesonide	30	47.09 ± 41.71	6.38 ± 1.40	5.31	< 0.001
ESR (mm/h)	Budesonide	24	58.46 ± 22.78	28.63 ± 7.68	8.42	< 0.001
	Ambroxol + budesonide	28	54.32 ± 23.78	22.86 ± 5.09	8.15	< 0.001
	Acetylcysteine + budesonide	30	52.30 ± 23.30	16.77 ± 2.66	8.72	< 0.001
LDH (IU/L)	Budesonide	24	587.58 ± 163.17	442.96 ± 138.87	7.96	< 0.001
	Ambroxol + budesonide	28	579.14 ± 153.18	354.36 ± 129.67*	13.16	< 0.001
	Acetylcysteine + budesonide	30	574.67 ± 136.54	211.57 ± 81.34*	19.95	< 0.001
WBC (10^9^/L)	Budesonide	24	9.95 ± 3.38	7.73 ± 2.01	3.48	< 0.002
	Ambroxol + budesonide	28	9.66 ± 3.24	7.82 ± 1.87	4.90	< 0.001
	Acetylcysteine + budesonide	30	9.38 ± 2.88	6.61 ± 1.22	7.14	< 0.001
SF (*u*g/L)	Budesonide	24	398.03 ± 60.80	202.75 ± 56.59	10.31	< 0.001
	Ambroxol + budesonide	28	394.43 ± 58.57	140.64 ± 21.04*	20.37	< 0.001
	Acetylcysteine + budesonide	30	399.33 ± 70.02	103.80 ± 20.24*	25.60	< 0.001

Annotate: * The changes in laboratory indices following therapy were compared across groups, and the difference in LDH and SF was statistically significant (X2 = 11.75, P < 0.05). Group Acetylcysteine + budesonide was superior to group ambroxol + budesonide (P0.05), whereas group budesonide was superior to group ambroxol + budesonide (P < 0.05)

### Pulmonary imaging changes

**Fig. 1:**
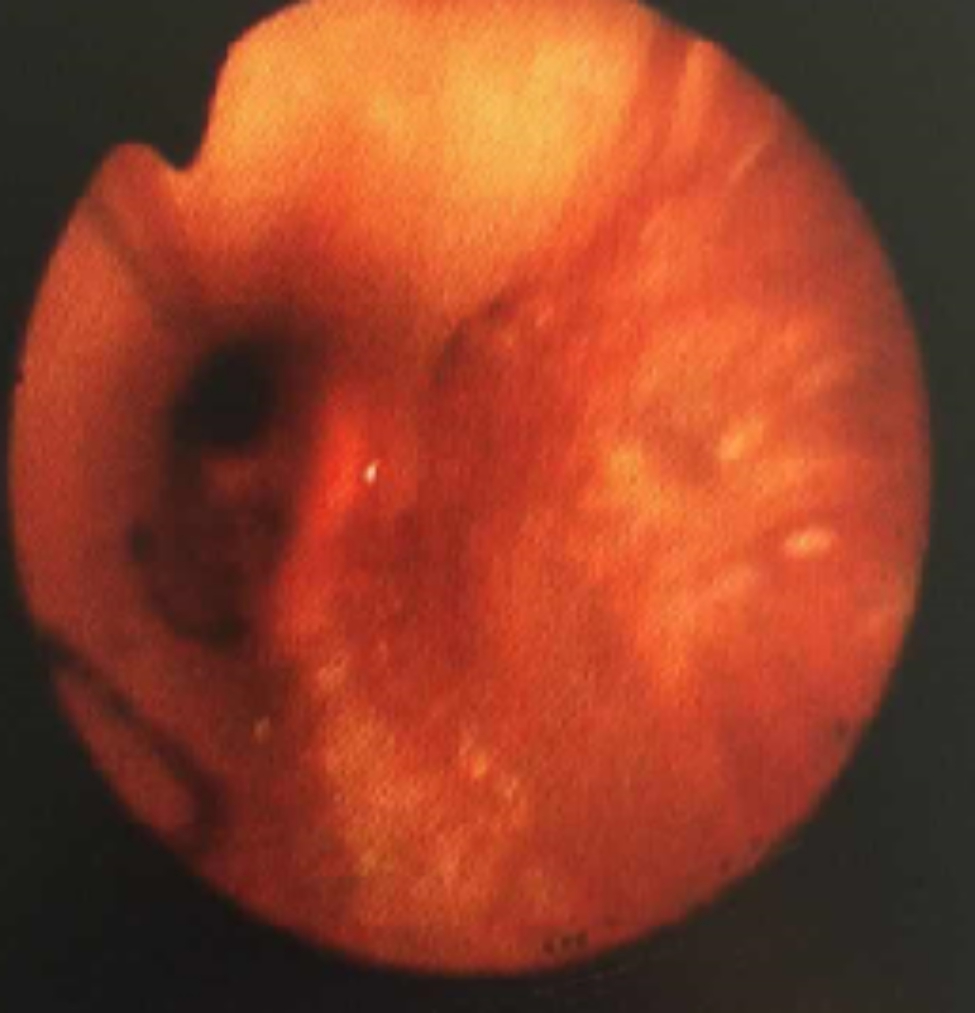
The right main airway was completely

**Fig. 2:**
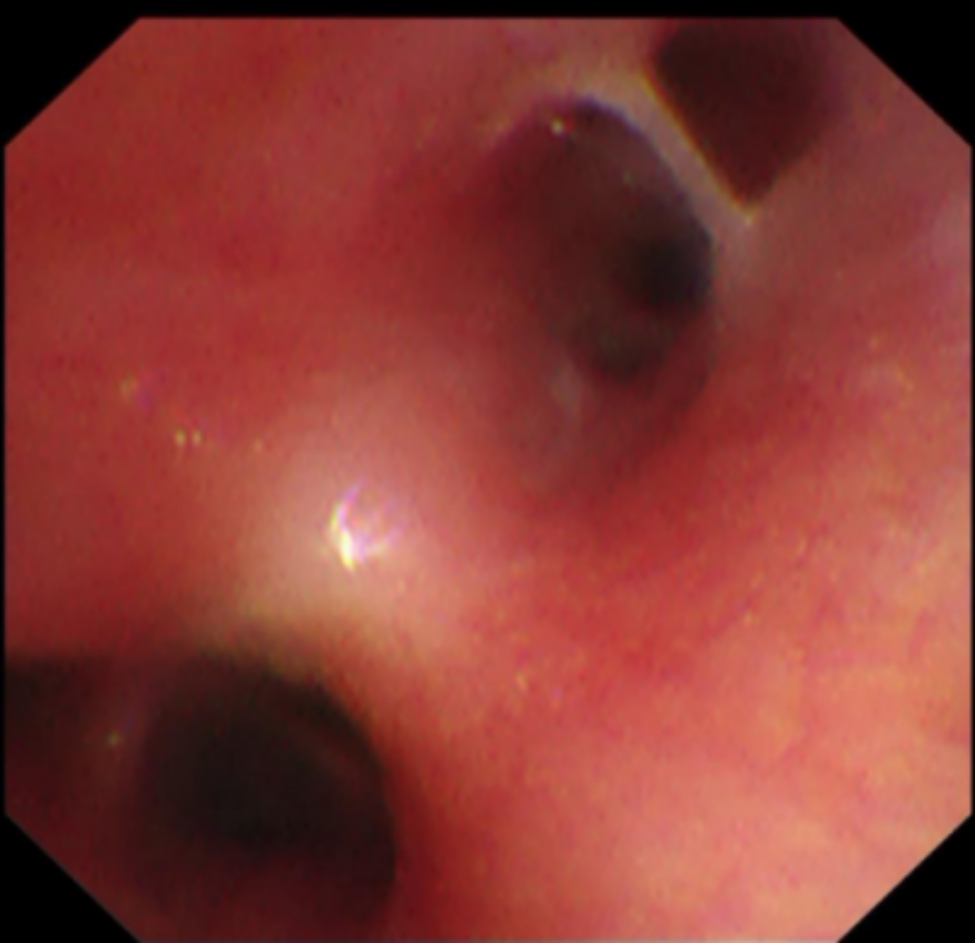
A mucous discharge originating from one branch of the bronchus

**Fig. 3:**
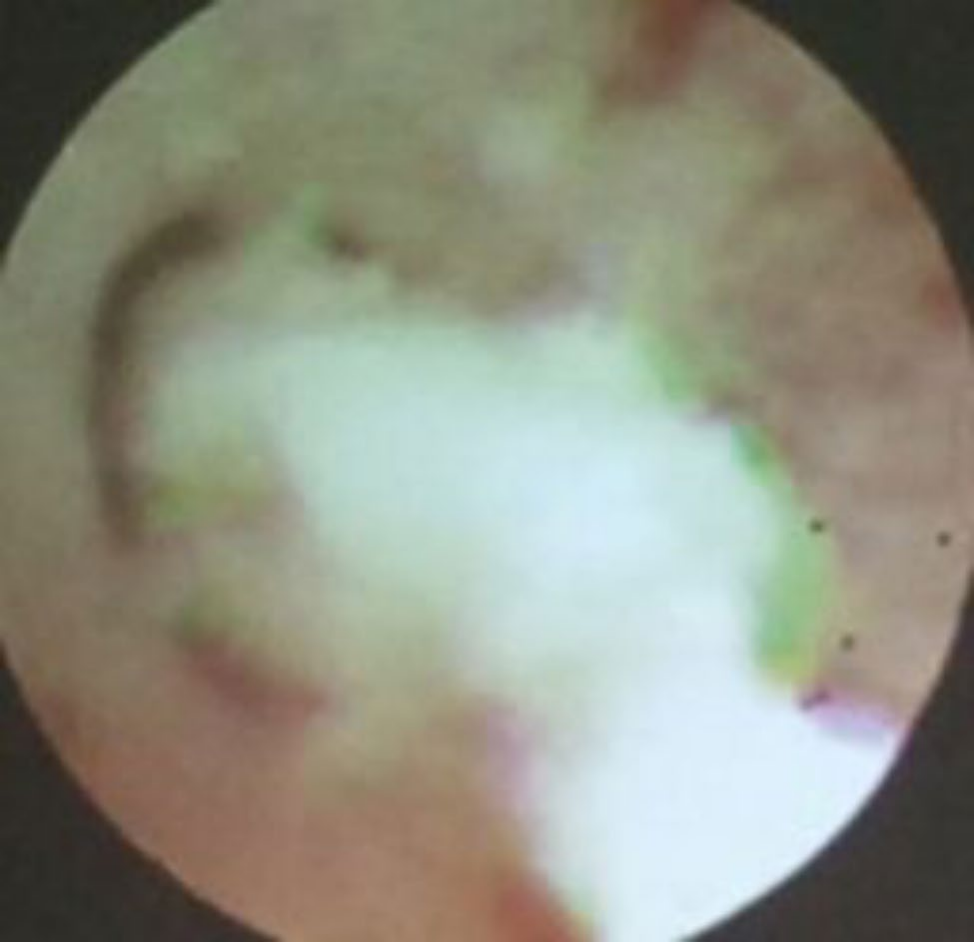
Sputum embolus

**Fig. 4:**
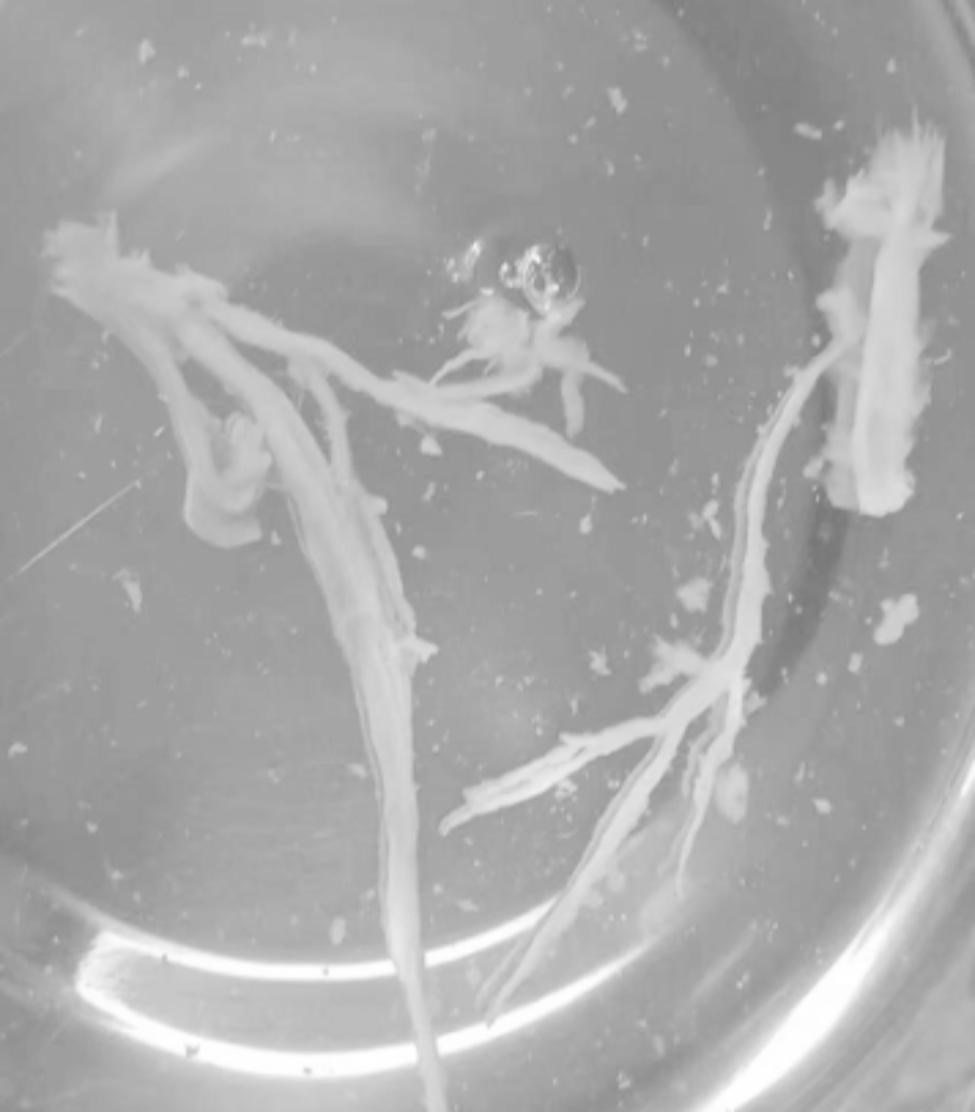
Sputum embolus was removed

**Fig. 5:**
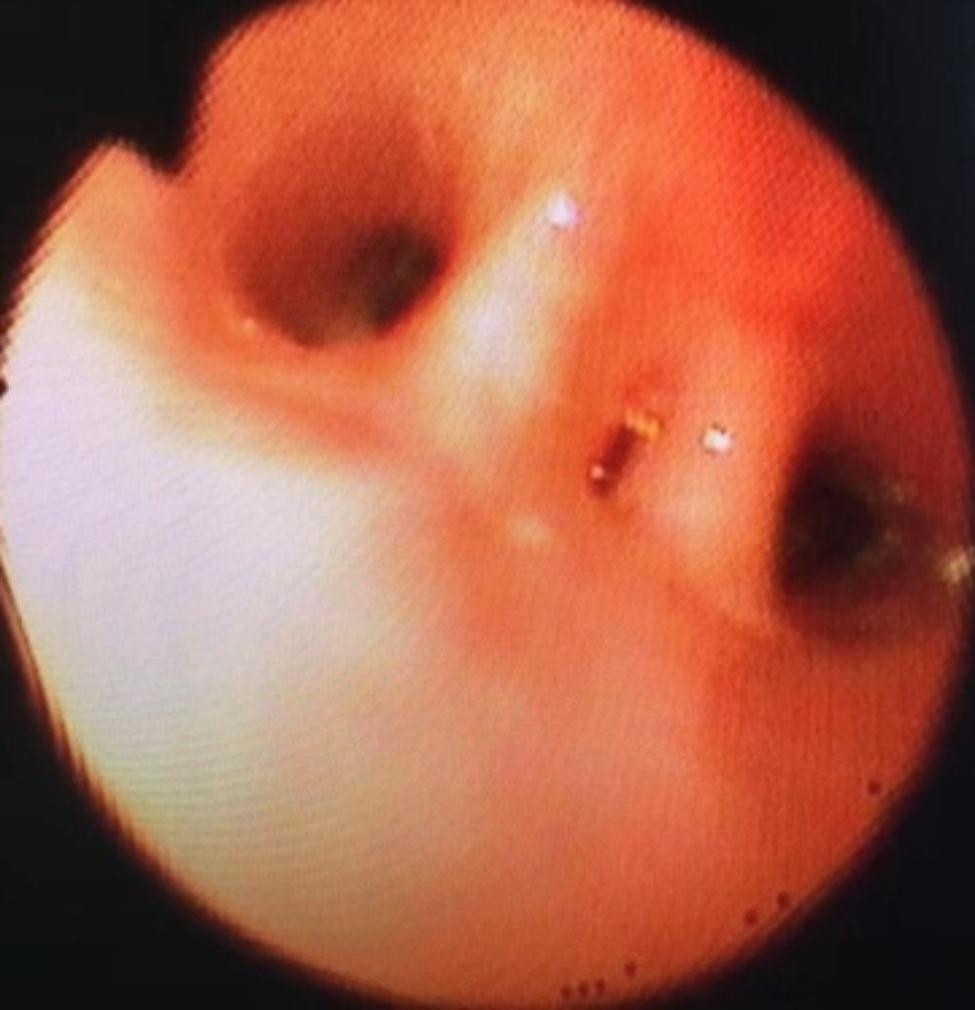
The mucosa of the bronchial wall was edema and stenosis

**Fig. 6:**
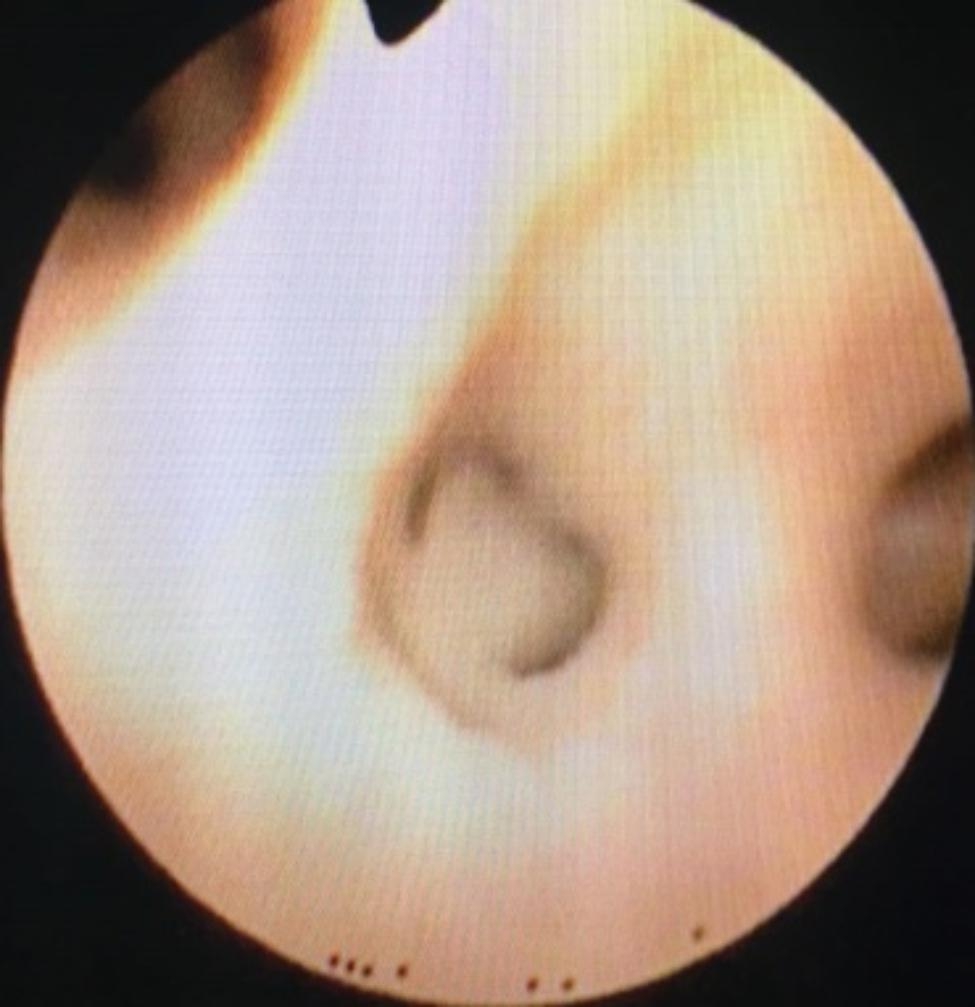
Sputum embolus

**Fig. 7:**
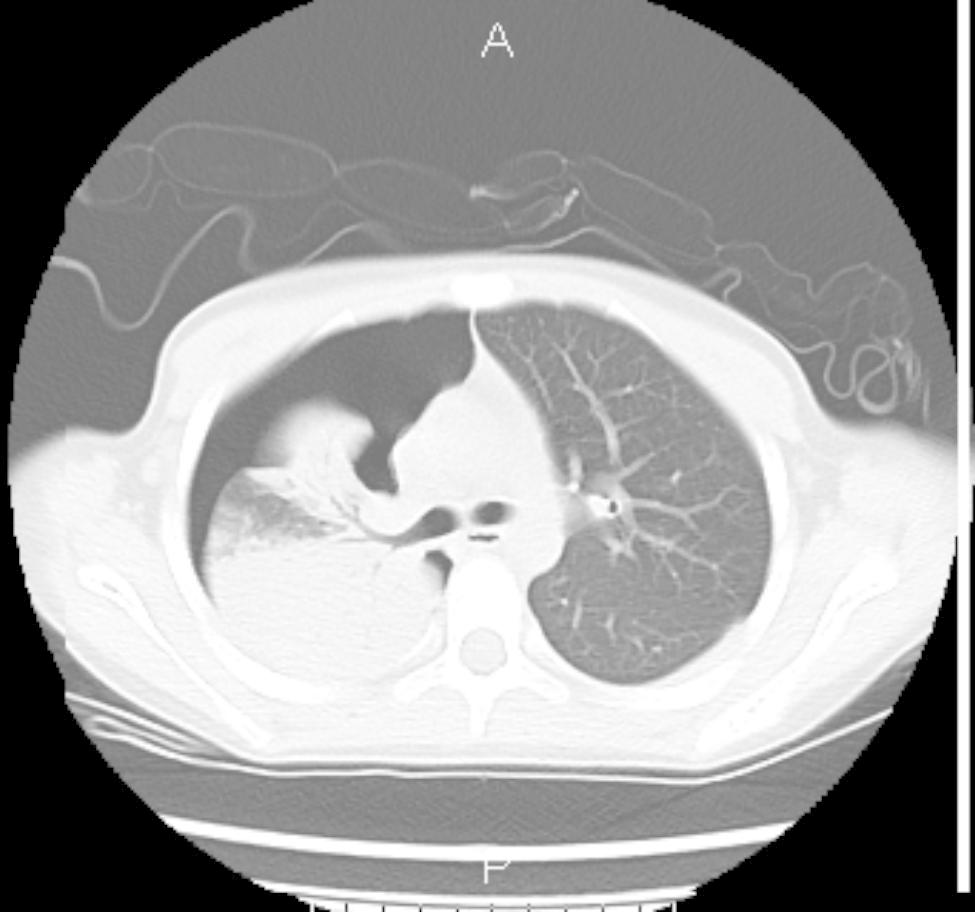
Pneumothorax lobar pneumonia

**Fig. 8:**
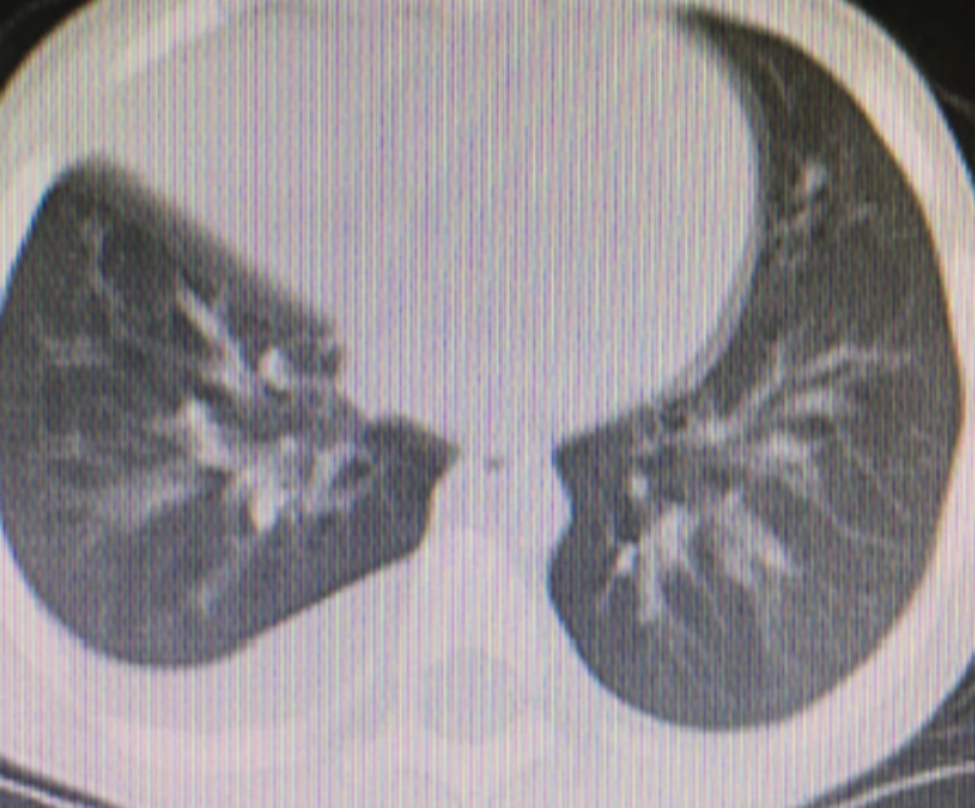
Pleuraleffusion Atelectasis

After one week of BLA therapy, the absorption area of lung lesions in the three groups differed significantly (X2 = 11.75, P > 0.05), and the improvement rate in the acetylcysteine + budesonide group was more significant than in the other two groups (P > 0.05). No significant differences existed between the remaining two groups (P > 0.05). Lung imaging one month after BLA therapy revealed that acetylcysteine + budesonide absorbed most inflammation. In 1 instance, however, there was no improvement in pulmonary imaging in either the budesonide or ambroxol + budesonide groups. After discharge, the clinical symptoms of one patient in the budesonide group improved, but pulmonary imaging revealed that bilateral lung inflammation had not been eliminated. Two months after combining BLA with ambroxol and budesonide twice, inflammation was eliminated. In the ambroxol + budesonide group, there were three incidences of atelectasis. One patient with atelectasis after BLA (with ambroxol + budesonide) was treated with BLA combination with acetylcysteine and budesonide atomization solution. Sputum embolus was extracted using pliers, followed by acetylcysteine injection and budesonide atomization inhalation. Three months following imaging of the lungs, bilateral pneumonia was entirely absorbed. After one week of BLA therapy, there was a significant difference between the three groups regarding lung imaging absorption. The differences between the three groups were statistically significant (X2 = 11.75, P > 0.05). However, there was no difference between the budesonide and ambroxol + budesonide groups (P > 0.05). Table 3; Figs. 1, 2, 3, 4, 5, 6, 7 and 8.

**Table 3:** Comparison of the pulmonary imaging changes after BLA in three groups with RMPP

Groups	cases	one week after BLA			one month after BLA			*X* ^*2*^	*P*
		Absorbed completely	Partial absorption	No change	Absorbed completely	Partial absorption	No change		
Budesonide	24	9	9	6	23	0	1	11.75	0.019
Ambroxol + budesonide	28	11	14	3	27	0	1		
Acetylcysteine + budesonide	30	22	7	1	30	0	0		

### Clinical efficacy comparison

There was a significant difference in clinical efficacy among the three groups. The clinical efficacy of the acetylcysteine + budesonide group was significantly superior to the other two groups (X^2^ = 9.78, *P <* 0.05), and there was no difference between the other two groups (*P*>0.05). Table 4.

**Table 4:** Clinical efficacy of three groups of children with RMPP

Groups	Cases	Remarkable (%)	Effective (%)	Invalid (%)	X^2^	*P*
Budesonide	24	33.33	41.67	25.00	9.78	0.044
Ambroxol + budesonide	28	35.71	42.86	21.43		
Acetylcysteine + budesonide	30	66.67	30.00	3.33		

### Comparison of the incidence of adverse reactions

The incidence of adverse reactions was 25% in the budesonide group, 21.4% in the ambroxol + budesonide group, 26.6% in the acetylcysteine + budesonide group, with no statistically significant difference between the three groups (*X*^2^ = 2.087, *P*>0.05).Table 5.

**Table 5:** Comparison of the incidence of adverse reactions in three groups of children with RMPP(%)

Groups	Cases	Transient hypoxemia		Mucosal hemorrhage		vomit	
		N	%	N	%	N	%
Budesonide	24	3	12.50	2	8.33	1	4.17
Ambroxol + budesonide	28	4	14.29	2	7.14	1	3.57
acetylcysteine + budesonide	30	4	13.33	3	10.00	1	3.33
		X^2^ = 2.087 *P*>0.05					

## Discussion

In recent years, an increasing number of Mycoplasma pneumoniae pneumonia (MPP) cases that are resistant, severe, and even fatal have been reported[11]. The airway and lung lesions of some individuals with refractory MPP (RMPP) remain severe, even if the children’s body temperature returns to normal after standard treatment. Confident children with RMPP suffer from problems, including atelectasis and pulmonary consolidation[12]. The bronchoscope has been widely used to diagnose and treat pediatric respiratory diseases. Among them, BLA fluid therapy is becoming in popularity [13]. The bronchoscope can reach the lesion directly during alveolar lavage, so not only can the secretions in the bronchus be aspirated, but also the drugs can be injected at the lesion, the treatment is targeted, which improves the clinical efficacy, and under the repeated flushing of alveolar lavage fluid, the mucous secretions can be diluted, which is more conducive to sputum discharge, effectively inhibit the toxin effect of pathogens,

Observation of clinical laboratory exams (WBC, CRP, ESR, LDH, SF), pulmonary imaging changes, clinical effectiveness, and the occurrence of adverse events before and after BLA with different drugs were the objectives of this research.

The overall time of bronchoscopic alveolar lavage therapy was shorter in the Acetylcysteine + budesonide group compared to the Budesonide and Ambroxol + budesonide groups, according to this research. Groups according to the overall length of treatment with various medicines. The difference between the budesonide and ambroxol + budesonide groups was not statistically significant. WBC, CRP, ESR, LDH, and SF levels decreased in all three groups after treatment, indicating that BLA may lessen the inflammatory response. The WBC, CRP, and ESR changes in the three groups after treatment were not statistically significant. The variations in LDH and SF, however, were statistically significant.

Tamura[14] observed that children with RMPP had elevated LDH and SF blood levels. Inamura et al.[15] believe that LDH, IL-18, and SF might be utilized to evaluate the therapeutic effectiveness of RMPP. Young-Jin Choi[16] revealed that LDH and SF might be a marker for children’s RMPP treatment. According to the results of this study, the effects of BLA coupled with acetylcysteine + budesonide were superior to those of BLA mixed with budesonide alone or with ambroxol + budesonide. In terms of reducing serum LDH and SF, acetylcysteine and budesonide’s effects are superior to those of cysteine and budesonide. The main component of acetylcysteine is N-acetyl-L-cysteamine Acid. Acetylcysteine may disrupt the disulfide bond of glycoprotein in sputum, prevent the activity of alveolar proteinase, break up mucus, and quickly dissolve viscous secretions. In addition, as an antioxidant, it may inhibit the generation of inflammatory factors, reduce the inflammatory response, and lessen mucosal stimulation, which is all useful for infection prevention. Some studies have shown that inhaled acetylcysteine is effective in reducing oxidative stress, and patients with higher oxidative stress may respond better to inhaled acetylcysteine therapy due to its ability to replenish glutathione and reverse the oxidant-antioxidant imbalance. Acetylcysteine is widely used to reduce sputum viscosity and stimulate expectoration. In China, the application of acetylcysteine in bronchoalveolar lavage is frequently reported. Furthermore, acetylcysteine and budesonide perfusion via fibrobronchoscope promote lesion recovery, alleviate pulmonary inflammatory response, and improve the therapeutic efficacy of refractory Mycoplasma pneumonia in children. The specific mechanisms of action are not currently clear.

Budesonide is a corticosteroid with vigorous glucocorticoid activity and modest halo-corticoid activity that aids in the relief of bronchial mucosal congestion and edema. It inhibits mast cells, eosinophils, neutrophils, macrophages, and lymphocytes, among other cells and mediators. In combination with acetylcysteine, local effects are faster than intravenous medicines, decreasing the side effects of systemic hormone therapy, and lung inflammation may be reduced. The unknown is the specific mechanism of action of serum LDH and SF. It is the active metabolite of bromhexine in the patient’s body, which ensures the mobility of mucus, improves the activity of tracheal cilia, reduces the secretion of sputum, and inhibits the accumulation of neutrophils, macrophages, leukotrienes, and other inflammatory cells and factors, thereby effectively reducing the inflammatory response of the body. During bronchoscopic alveolar lavage, it has been demonstrated to be inferior to acetylcysteine in decreasing inflammation. Further research is required to determine if this is because acetylcysteine is a nebulized medication with a low molecular weight, which makes it more likely to have an anti-inflammatory impact.

According to the results of this study, the clinical efficacy of combination lavage with acetylcysteine + budesonide was superior to that of budesonide alone or ambroxol + budesonide. The atomized dose form of acetylcysteine makes it easier to reduce sputum viscosity, dilute sputum, and make sputum easier to cough up than the intravenous infusion form of ambroxol injection. As a flushing solvent, ordinary saline was incapable of lowering sputum viscosity.

It is the active metabolite of bromhexine in the patient’s body, which ensures the mobility of mucus, improves the activity of tracheal cilia, reduces the secretion of sputum, and inhibits the accumulation of neutrophils, macrophages, leukotrienes, and other inflammatory cells and factors, thereby effectively reducing the inflammatory response of the body. During bronchoscopic alveolar lavage, it has been demonstrated to be inferior to acetylcysteine in decreasing inflammation. Further research is required to determine if this is because acetylcysteine is a nebulized medication with a low molecular weight, which makes it more likely to have an anti-inflammatory impact.

Cough was the most common adverse reaction to BLA, followed by tachycardia or bradycardia, mild bleeding, bronchospasm, laryngeal spasm, dyspnea, sore throat, and decreased blood oxygen [17].

According to specialized studies, the rate of adverse events ranges from 5 to 35%, although serious adverse events are below 1% [18, 19]. According to this research, there was no significant difference in the occurrence of adverse responses between the three groups. The cough was the most prevalent side effect of BLA therapy, followed by an elevated heart rate. These side effects subsided after BLA was withdrawn, demonstrating that the combination of BLA and the other three medications was safe.

This research indicated that BLA might boost the early therapeutic Impact of RMPP, efficiently eliminate pathogens and thick respiratory tract secretions, as well as sputum embolism, consequently improving the recovery of small airway structure and function and decreasing hospital stays. BLA is affected differently by different drugs. BLA-coupled acetylcysteine and budesonide were more effective than ambroxol combined with budesonide and budesonide alone; hence, they are clinically applicable.

## Conclusions

BLA-coupled acetylcysteine + budesonide was superior to the other two groups in enhancing the effectiveness of RMPP in children, which might increase lung opacity absorption and minimize lung inflammation. Insufficient sample size and potential data collection omissions indicate the necessity of conducting large-scale, multicenter studies.

## Data Availability

The datasets used and/or analyzed during the current study are available from the corresponding author on reasonable request.
